# Label-Free Surface-Enhanced Raman Spectroscopy with Machine Learning for the Diagnosis of Thyroid Cancer by Using Fine-Needle Aspiration Liquid Samples

**DOI:** 10.3390/bios14080372

**Published:** 2024-07-31

**Authors:** Lili Gao, Siyi Wu, Puwasit Wongwasuratthakul, Zhou Chen, Wei Cai, Qinyu Li, Linley Li Lin

**Affiliations:** 1 Department of Pathology, Ruijin Hospital, Shanghai Jiao Tong University School of Medicine, 197 Ruijin Second Road, Shanghai 200025, China; gll12216@rjh.com.cn; 2 School of Biomedical Engineering, Shanghai Jiao Tong University, Shanghai 200030, Chinachenzhou96@sjtu.edu.cn (Z.C.); linli92@sjtu.edu.cn (L.L.L.); 3 Department of General Surgery, Ruijin Hospital, Shanghai Jiao Tong University School of Medicine, 197 Ruijin Second Road, Shanghai 200025, China; caiwei@shsmu.edu.cn

**Keywords:** Raman spectroscopy, thyroid fluid, machine learning, CNN, liquid biopsy

## Abstract

The incidence of thyroid cancer is increasing worldwide. Fine-needle aspiration (FNA) cytology is widely applied with the use of extracted biological cell samples, but current FNA cytology is labor-intensive, time-consuming, and can lead to the risk of false-negative results. Surface-enhanced Raman spectroscopy (SERS) combined with machine learning algorithms holds promise for cancer diagnosis. In this study, we develop a label-free SERS liquid biopsy method with machine learning for the rapid and accurate diagnosis of thyroid cancer by using thyroid FNA washout fluids. These liquid supernatants are mixed with silver nanoparticle colloids, and dispersed in quartz capillary for SERS measurements to discriminate between healthy and malignant samples. We collect Raman spectra of 36 thyroid FNA samples (18 malignant and 18 benign) and compare four classification models: Principal Component Analysis–Linear Discriminant Analysis (PCA-LDA), Random Forest (RF), Support Vector Machine (SVM), and Convolutional Neural Network (CNN). The results show that the CNN algorithm is the most precise, with a high accuracy of 88.1%, sensitivity of 87.8%, and the area under the receiver operating characteristic curve of 0.953. Our approach is simple, convenient, and cost-effective. This study indicates that label-free SERS liquid biopsy assisted by deep learning models holds great promise for the early detection and screening of thyroid cancer.

## 1. Introduction

The global incidence of thyroid cancer, characterized by the malignant proliferation of cells within the thyroid gland, has been on the rise [1]. Thyroid cancer is one of the most common endocrine malignancies, with its incidence increasing rapidly worldwide [2]. The early and accurate diagnosis of thyroid cancer is crucial for effective treatment and improved patient outcomes, making it a significant public health concern.

Fine-needle aspiration (FNA) cytology of the thyroid is the initial screening test and a widely utilized diagnostic technique for thyroid nodules. FNA involves the extraction of biological samples containing cell clusters from the lesion for further analysis [2], offering a less invasive alternative compared to incisional or excisional biopsy. Samples collected by FNA are further used for FNA cytology: either applied directly for cell smear cytology or transferred to the preservation solution for liquid-based cytology slide preparation. The remaining FNA samples are washed out and stored in the preserving solution, denoted as FNA washout fluids (or FNA washouts).

FNA cytology involves spreading cells on slides for staining and the microscopic examination of the overall morphology of each cell. At present, FNA cytology is the gold standard for malignant or benign cells examination. However, this process is labor-intensive and relies heavily on the expertise of specialists. In addition, FNA cytology can lead to the risk of false-negative diagnoses if the number of cells punctured is insufficient or the slide staining is unsatisfactory. For example, according to the Bethesda System for Reporting Thyroid Cytopathology, non-diagnostic or unsatisfactory FNA cytology carries a 5–10% risk of malignancy [3]. Although molecular-level biological information such as genomic profile can help in the accurate classification of thyroid nodules, these methods are too expensive, time-consuming, requiring a central laboratory; none have achieved the reliability required for routine clinical diagnosis [4]. Therefore, the development of a rapid and accurate diagnostic method using FNA samples is of great importance.

A strategic way to achieve this goal is Raman spectroscopy. This technique is non-destructive on analytes and provides rich fingerprint information by probing the vibrational and transitional dynamics of the chemical bonds. It can be used to obtain molecular information from cellular components such as proteins, lipids, and DNA, and has great potential in pathological evaluation [5,6,7]. For these characteristics, it has been applied on a variety of biological samples to differentiate between healthy and cancer tissues [8,9,10], such as thyroid cancer diagnosis [11,12,13,14]. Machine learning (ML) is effective in analyzing complex, large, high-dimensional data sets [15,16,17], advantageous for analyzing Raman spectral information [18,19]. At present, the application of ML algorithms to classify Raman spectra for diagnostic purposes has shown great potential, allowing the successful recognition of biochemical changes associated with oncogenesis in cancers of the digestive system [20,21,22], lung [23,24], thyroid gland [25], etc.

Surface-enhanced Raman spectroscopy (SERS) can amplify the Raman signal of analytes by over 10 times when the analytes are adsorbed on plasmonic metal nanostructures [26,27]. Therefore, SERS has become a promising bioanalytical technology for a wide range of cancers and diseases [28,29,30]. SERS substrates have been used for thyroxine quantification [31], the selective detection of human Thyroglobulin [32], etc. Particularly, label-free SERS is quite suitable for liquid sample detection by utilizing gold (Au) or silver (Ag) colloids as substrates. This technique is known as SERS liquid biopsy. Also, the combination of label-free SERS with ML algorithms has been explored for thyroid diagnosis. For example, SERS detection using Ag nanowire membrane combined with multiple machine learning algorithms has been reported to analyze FNA extracted tissues to diagnose malignant and benign thyroid nodules with an accuracy of 95.59% [33]. Wang et al. presented the label-free SERS profiling of exosomes in serum using Au@Ag substrates combined with deep learning to distinguish thyroid cancer patients from healthy controls with 96% accuracy [34]. For Raman-based thyroid diagnosis, tissues [25,35] and cells derived from FNA tissue fragments [36,37], serum [38,39], or plasma [40] are commonly used; while FNA washout fluids for label-free SERS have not been fully explored [14]. The FNA washouts contain cell clusters and biomarkers derived from extracellular and intracellular environments. The SERS profiling of FNA washouts, coupled with advanced ML algorithms, is thus anticipated for the accurate and rapid diagnosis of thyroid cancer.

**Figure 1:**
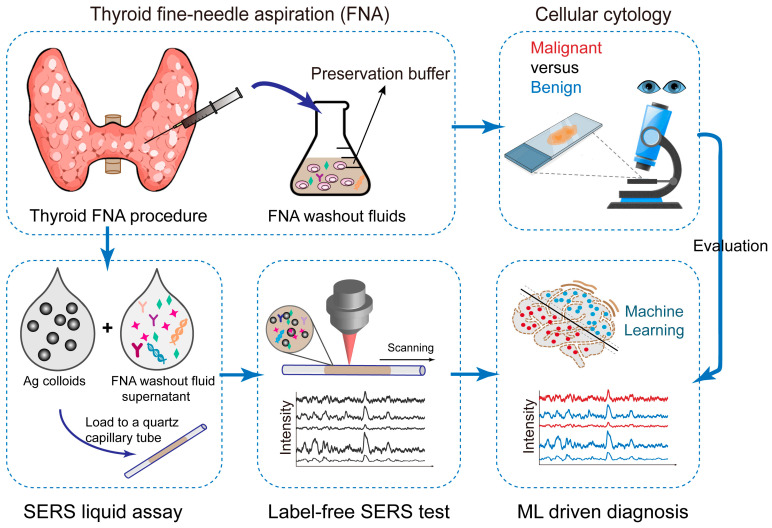
Scheme of the work flow. FNA samples are first used for cell cytology, as the gold standard diagnosis. Then, excess FNA samples are stored in the preservation phase as the FNA washout fluids. The fluid supernantant is mixed with Ag colloids for SERS enhancement, and this liquid mixture is loaded into microfluidic quartz capillaries for Raman measurements. We collected the Raman spectra for machine learning classification. Cell cytology results are used for evaluation of SERS diagnosis accuracy.

In this study, we develop a label-free SERS liquid biopsy technique supported by ML to achieve the rapid and accurate diagnosis of thyroid cancer by using thyroid FNA washout fluids (Figure 1). We choose Ag nanoparticle colloids as SERS substrates because they are widely used with good reproducibility in a low-cost and scalable synthetic manner. FNA washout fluids in the preservation phase are mixed with Ag colloids for SERS enhancement. This liquid mixture is then loaded into microfluidic quartz capillaries for Raman measurements. We collected the Raman spectra of 36 clinical samples (18 malignant/18 benign) and compared four classification models: Principal Component Analysis–Linear Discriminant Analysis (PCA-LDA), Random Forest (RF), Support Vector Machine (SVM), and Convolutional Neural Network (CNN). Our results showed that the CNN algorithm is the most stable and has the highest accuracy, sensitivity, and precision. This study indicates that ML-assisted label-free SERS using the FNA fluids holds great promise for the early detection and screening of thyroid cancer. This approach is simple, convenient, minimally invasive, and cost-effective, providing valuable insights for non-invasive and rapid clinical diagnosis.

## 2. Materials and Methods

### 2.1. Materials

Sodium citrate (≥98%), silver nitrate (AgNO_3_, 99.8%), and sodium chloride (NaCl, ≥99.5%) were received from Sinopharm Chemical Reagent Co., Ltd. (Shanghai, China). The ascorbic acid (>99.0%) was obtained from Aladdin (Shanghai, China). Nanopure water was used for all the experiments. All the materials were used as received without any further purification.

### 2.2. Preparation of Ag NPs

Citrate-stabilized Ag colloids were synthesized according to our established method based on a previous report [7]. First, 15 μL of AgNO_3_ aqueous solution (1 M) was added to 2 mL of sodium citrate aqueous solution (1 wt%) while stirring, and the mixture was incubated in darkness at room temperature for 5 min. Then, 3 μL of sodium chloride aqueous solution (2 M) was added. Separately, 50 μL of ascorbic acid aqueous solution (1 M) was added to 100 mL of boiling water and the initial solution was added in. The mixture rapidly changed from colorless to yellow, and then kept boiling with stirring for 1 h. The colloids could be synthesized in large batches and stored at 4 °C for further use. The theoretical concentration of the obtained Ag NPs was approximately 0.2 nM according to our previous report [41]. The Ag colloids were concentrated 20-fold by centrifugation, making the final concentration 4 nM.

The UV–vis extinction spectra of Ag colloids were measured from a UV1900 UV–vis spectrophotometer (Aucybest, Shanghai, China). TEM images were collected from a JEM-2100F Transmission Electron Microscopy (JEOL, Tokyo, Japan).

### 2.3. Clinical Sample Collection and Storage

The study cohort comprised patients diagnosed with thyroid nodules who underwent FNA procedures at Ruijin Hospital, School of Medicine, Shanghai Jiao Tong University. Ethical approval was obtained from the institutional review board, and informed consent was obtained from all the participants. The FNA samples were collected from each patient. After the usage for cell cytology, the remaining FNA samples were washed out and stored in the preserving solution as FNA washout fluids for our study.

A total of 36 thyroid FNA washout fluid samples (18 malignant and 18 benign) were collected from 18 patients. The cohort consisted of 14 females and 4 males, reflecting the higher incidence of thyroid cancer in women. The cohort was with an age distribution as follows: 9 patients between 25 and 45 years, and 9 patients over 45 years. All the patients included in this study were in the early stages of thyroid cancer, with no metastasis observed. The FNA cytology was used as the diagnosis gold standard according to the FNA cytology examination based on the Bethesda system diagnostic categories [3]. The malignant samples were all papillary carcinoma; the benign samples were benign follicular nodules or healthy cells collected from the same thyroid nodules.

### 2.4. Labe-Free SERS Measurements on FNA Washout Fluids

**Figure 2:**
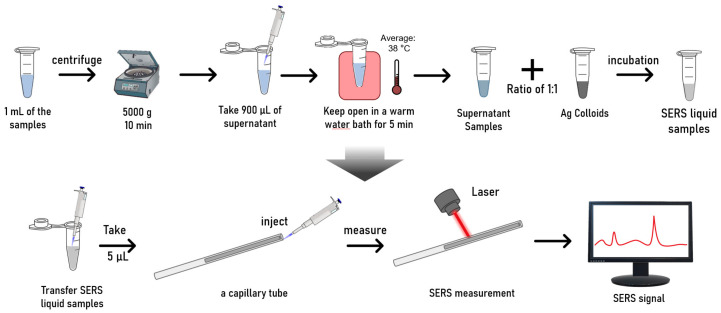
Workflow of the sample preparation and SERS measurements.

The workflow is shown in Figure 2. Before use, 1 mL of FNA washout fluid sample was taken out from the refrigerator and allowed to stay at room temperature for 10 min. The samples were then centrifuged at 5000× *g* for 10 min to separate the cellular components. After centrifugation, the sediment, mainly consisting of cells, was discarded, and the supernatant (900 µL) was carefully transferred to a new tube for further use.

Next, the supernatant samples were left open for 5–10 min to allow some volatile substances in the preservation solution to evaporate. Mild heating helps accelerate this process. Therefore, we placed the samples in an open container in a warm water bath for 5 min. During the mild heating process, the temperatures of the samples were around 38 °C. This temperature range is below the temperature that typically causes protein denaturation, therefore, a safe temperature, particularly with a short incubation time. This step can help reduce the Raman background interference of volatile substances in the preservation solution.

After the above pre-treatment process, the supernatant was mixed with concentrated Ag colloids in a 1:1 volume ratio. To ensure a thorough mixing of the NPs and biological molecules, the mixture was sonicated for several seconds in the sonication bath. The mixture was then incubated undisturbed at room temperature for 30 min. This process aimed to provide sufficient interaction time between the sample molecules and Ag NPs.

For the SERS measurements, the mixture was loaded into a microfluidic quartz capillary (inside diameter: 1 mm, outside diameter: 2 mm) and placed on the sample stage of a confocal Raman spectrometer (XploRA INV, Horiba, Kyoto, Japan). We loaded 5 µL of the liquid mixture, forming a liquid column inside the tube. The quartz tube was fixed on the sample stage, and the objective lens was adjusted to focus the laser onto the quartz tube containing the liquid sample. Using a 638 nm laser with an acquisition time per spectrum of 1 s (60× objective, 25% of full laser power, Raman shift range: 300–2000 cm^−1^). Spectra were acquired from the mixture (Figure 2). During the measurements, we moved the sample stage in the x-y direction to excite and collect SERS signals from different locations within the quartz tube. A mapping step size of 15–20 µm was applied between adjacent measuring points. In this manner, multiple spectra (n = 100) were collected for each sample at various locations within the quartz tube. In total, we obtained 1800 spectra for the malignant and benign thyroid FNA samples, respectively.

### 2.5. Raman Data Processing and Machine Learning Classification Model

Spectral noise reduction was applied using the Savitzky–Golay filter (with a window size of 5, and an order of 3). We adopted the adaptive iteratively reweighted penalized least squares (airPLS) method (order of 3 and lambda of 150) to perform baseline correction for each spectrum. For further analysis, SERS spectrum data were linearly normalized to the range of (0–1).

**Figure 3:**
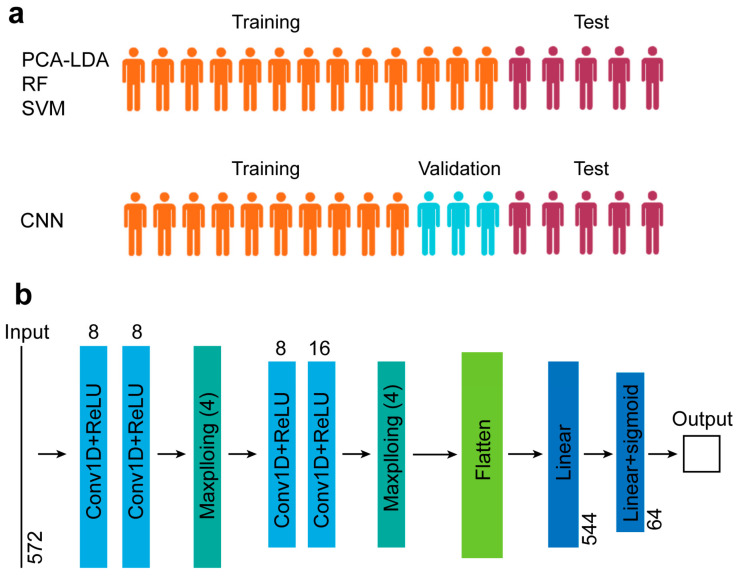
(**a**) Group division. (**b**) CNN architecture and hyperparameters.

We established four classification methods, including PCA-LDA, RF, SVM, and CNN. We separate the patients into the training, validation, and test sets in the ratio of 10:3:5. For the PCA-LDA, RF, and SVM models, the validation and training sets were combined as a training set, i.e., the ratio is 13:5 for the training and test sets, respectively (Figure 3a). All the groups were divided at the patient level, i.e., the spectra from one sample were included in one group.

For PCA-LDA, LDA was performed using the 12 score vectors of PCA, explaining 95% of the variation in the training set. For the RF analysis, the maximum number of estimators is 200. Raman spectra were divided into training and test sets in a 2:1 ratio at the patient level for the RF and SVM models. The network architecture of our CNN comprises a stack of convolutional layers with ReLU activation, max-pooling layers, fully connected layers, and a sigmoid layer (see more details in Figure 3b). Our model uses Adam for optimization [42]. The initial learning rate is 0.001, which decays with a factor of 0.5. The model is trained with a batch size of 64. The maximum epoch number is 50. The final model is selected where the validation loss is the minimum. Our objective function uses binary cross entropy. All the spectral preprocessing and classification algorithms were completed using Python 3.9. The calculations are implemented based on one NVIDIA GeForce RTX 3090 GPU (ThinkStation, Lenovo, China).

### 2.6. Classification Model Performance Analysis

We used different metrics to evaluate the performance of the classification models, namely sensitivity, specificity, and accuracy, which are defined with the following formulas:

(1) $$Sensitivity=\frac{TP}{TP+FN}$$

(2) $$Specificity=\frac{TN}{TN+FP}$$

(3) $$Accuracy=\frac{TP+TN}{TP+TN+FP+FN}$$

where TP, FN, TN, and FP are the number of true positive, false negative, true negative, and false positive samples, respectively. They are shown in the confusion matrix.

The receiver operating characteristic (ROC) curve, as a comprehensive indicator of the sensitivity and specificity of the algorithm, is often used to evaluate the accuracy and stability of the models. ROC is a graphical analysis tool, and the vertical axis represents the true positive rate (sensitivity), and the horizontal axis represents the false positive rate (1-specificity). The area under the curve (AUC) is used as an indicator of the scoring model. The closer the AUC is to 1, the better the algorithm works.

## 3. Results

### 3.1. SERS Liquid Assay Optimization and Sample Pre-Treatment

**Figure 4:**
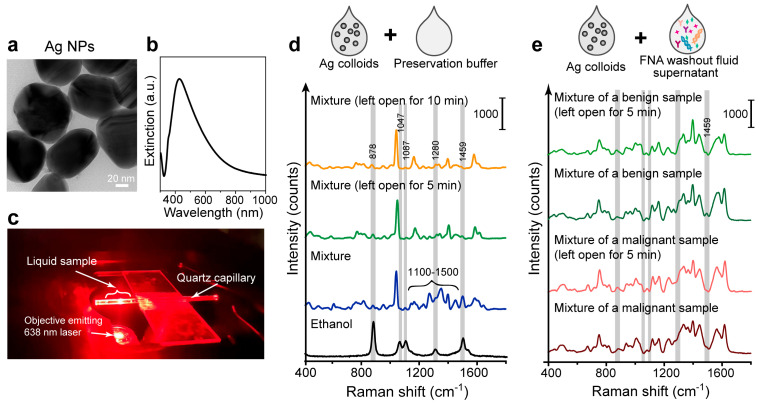
Label-free SERS measurements using the Ag colloids and thyroid FNA washout liquids. (**a**) TEM image and (**b**) UV-Vis spectra of the Ag colloids. (**c**) Picture of the SERS measurement setup. (**d**) SERS measurements of the pure preservation buffer mixed with Ag colloids. The mixture was measured without or with open-air exposure for 5 or 10 min with mild heating. The spectrum of pure ethanol (black curve) is for comparison. (**e**) SERS measurements of the FNA washout fluid supernatant mixed with Ag colloids. Representative spectra of a benign or a malignant sample were displayed. The mixture was measured without exposure or after 5 min open-air exposure with mild heating.

In this work, we applied the SERS measurements in the liquid phase using the Ag colloids as plasmonic substrates. The liquid SERS method improves the uniformity and convenience of sample collection and measurement. This leads to more consistent and reproducible measurements compared to spreading liquid samples on a slide where uneven spreading and drying can affect the SERS signal. To begin with, we prepared the citrate-stabilized Ag colloids. They have a diameter of ~45 nm in TEM images (Figure 4a) and the localized surface plasmon resonance absorbance peak at ~420 nm (Figure 4b). The typical measurement setup is shown in Figure 4c: the pre-processed FNA samples were mixed with Ag NP colloids and loaded into a quartz capillary tube for SERS measurements using a 638 nm laser. In the liquid-phase environment, examining the distribution of NPs alone is less meaningful because we are concerned with the overall situation rather than individual hot spots. Instead, we aimed to obtain a relatively uniform signal through the collection and averaging of multiple repeated SERS spectra to enhance consistency and productivity. Averaging SERS spectra over a large number of single-sample spectra (e.g., 100 spectra) helps mitigate the impact of outliers and random fluctuations, as reported in our previous studies [43]. Therefore, we scanned the capillary tube and collected 100 spectra for each sample.

The components of the commercial preservative buffers vary, but most commonly include formaldehyde, glutaraldehyde, and ethanol, some of which are volatile organic compounds. To assess the background interference of the preservative buffer, its SERS spectrum was first tested. We measured the SERS spectrum of the preservative buffer mixed and incubated with Ag colloids. The spectral data of 100 individual spectra per sample were averaged to obtain the mean spectrum. As shown in Figure 4d (blue curve), the SERS spectrum of the mixture exhibited many small, cluttered peaks, particularly in the 1100–1500 cm^−1^ range. We then left some of the buffer open for 5 or 10 min before mixing with the Ag colloids, allowing the evaporation of volatile substances with this treatment; the SERS spectrum of buffer showed fewer peaks in the 1100–1500 cm^−1^ range, resulting in a cleaner background (Figure 4d, blue and green curve). The SERS spectrum after the treatment also indicated a reduction in ethanol peaks, especially around 1459 cm^−1^, when compared with the pure ethanol spectrum (Figure 4d, black curve). This result suggests that open-air exposure can help reduce the Raman background interference of volatile substances in the preservation solution. We would like to note that we adopted mild heating (up to 38 ± 2 °C, see Section 2) that potentially helps accelerate this process, while this process can also be carried out at room temperature with a longer duration. As the spectral differences between 5 and 10 min of open-air exposure with mild heating were minimal (Figure 4d, blue and green curve), we finally adopted the shorter pre-treatment time, i.e., 5 min open-air exposure with mild heating.

To confirm that this pre-treatment process effectively reduces the preservative buffer background without affecting the biological molecules in the samples, we applied it to the real FNA washout fluid samples for SERS detection. The FNA samples were centrifuged to remove cellular debris, and the supernatant was mixed with Ag colloids and incubated for SERS measurements (see Section 2). We compared the SERS spectra of mixtures subjected to 5 min open-air exposure versus direct testing, displaying representative spectra for a benign and a malignant sample. Averaging the SERS spectra over 100 spectra of a single sample was also adopted to mitigate the impact of random fluctuations. As we can see in Figure 4e, there is a slight reduction in ethanol peak around 1459 cm^−1^, and subtle changes in peak ratios between 1100 and 1500 cm^−1^, confirming the evaporation of volatile substances. Overall, this pre-treatment process had minimal effect on the overall spectral shape. Thus, this pre-treatment process is deemed appropriate for testing FNA fluids.

### 3.2. Acquisition of SERS Spectra on Thyroid FNA Washout Biofluid Samples

**Figure 5:**
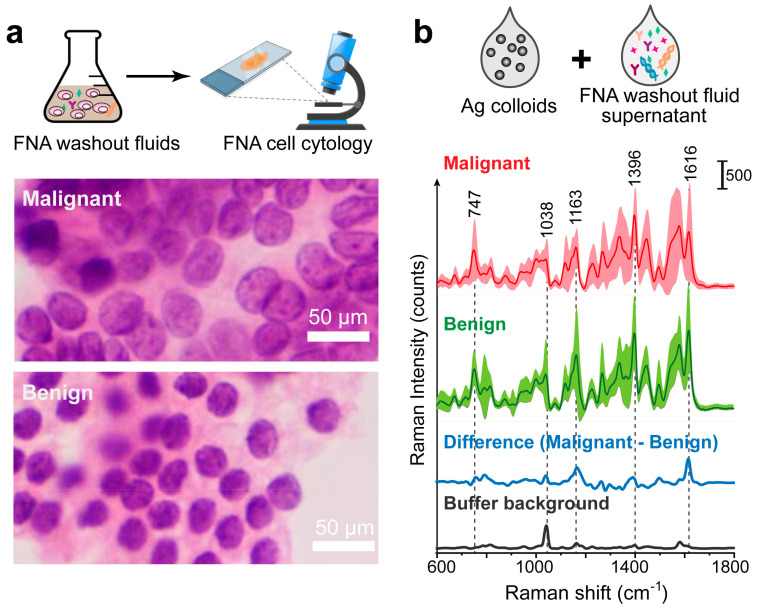
FNA cytology results and label-free SERS measurements of FNA washout liquids. (**a**) FNA cell cytology image. (**b**) Averaged SERS spectra with standard deviations of thyroid malignant and benign samples, as well as the averaged difference spectrum (malignant–benign). This is the label-free SERS measurements of the FNA washout liquids mixed with Ag colloids. Standard deviation values are shown in shadow. The SERS spectrum of the preservation buffer is also displayed (black curve).

We continued to use thyroid FNA washout fluids samples for SERS examination, as shown in the workflow (Figure 5). Here, FNA cell cytology images were used as a gold standard assessment of malignant or healthy cells. We display the malignant papillary thyroid carcinoma (Figure 5). This monolayer sheet with a syncytial-like appearance comprises cells with irregular nuclei that show focal molding. Compared to the image of benign follicular cells (Figure 5a), they have round to oval, monomorphic nuclei with finely granular chromatin and inconspicuous or absent nucleoli.

The FNA washouts were centrifuged to remove precipitates, mainly the cells, and the supernatant was mixed with Ag colloids and incubated for 30 min before SERS measurements. In total, we obtained 1800/1800 spectra for the malignant/benign thyroid FNA samples, respectively. We aimed to obtain a relatively uniform signal by collecting and averaging multiple SERS spectra. This emphasizes the common vibrational bands characteristic of the sample types, providing a clearer basis for distinguishing between malignant and benign samples. In Figure 5b, we investigated the averaged SERS spectra of the malignant and benign groups (red and green curves). The average spectrum for each group was calculated by averaging the mean spectra of all samples within the group.

The SERS spectrum of the cancerous and benign FNA samples share similar peak positions but different intensities or peak ratios, which reflects the contributions of biochemical molecules in the progression of thyroid cancer. For example, the Raman peaks at 1163 cm^−1^ could be attributed to C-C skeletal stretch transconformation, related to lipids and proteins [14]. According to the normal Raman spectrum of thyroid carcinoma, the band at 747 cm^−1^ is strong and could be assigned to the fingerprint of cytochrome c, a protein localized in the mitochondrial intermembrane space [13,14]. We notice that the preservation buffer also has some characteristic peaks, with the main band at around 1036 cm^−1^ (Figure 5b, black curve). This may overlap with that of the test sample. But the good thing is that, since the preservation buffer is used as a solution, this peak serves as a constant background present in all SERS spectra. Thus, we can still compare the differences between healthy and malignant groups.

The averaged difference spectrum was conducted by subtracting the mean spectrum of the benign group from the malignant group at each Raman shift (Figure 5b, blue line) for better comparison between the two groups. In the difference spectrum, there is a clear band position around 1038 cm^−1^, which could be attributed to the τ(HCH) mode, rich in collagen, phospholipids, and phenylalanine [14]. The peak at 1616 cm^−1^ is possibly due to the presence in the sample of amide I and lipids [13], and the 1396 cm^−1^ band could be assigned to CH-rocking [35], perhaps related to carbohydrates [14]. The main SERS band positions and their potential assignment to biochemical components are listed in Table 1. Overall, these distinct differences in SERS spectra show the potential for the Raman diagnosis of thyroid tumors.

### 3.3. Classification Model on SERS Spectra

**Figure 6:**
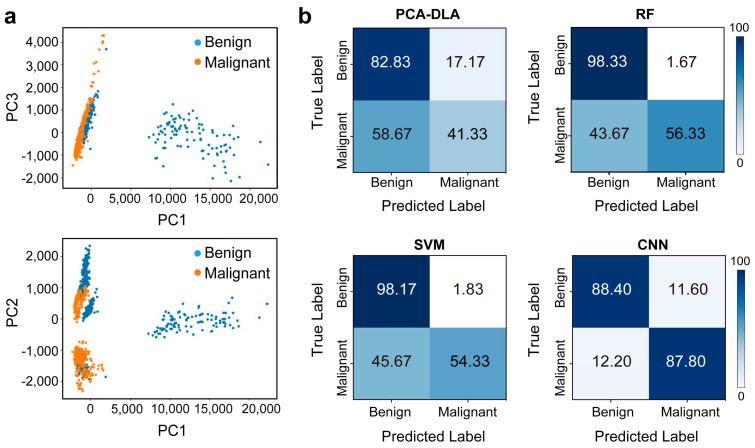
(**a**) PCA clustering results. (**b**) Confusion matrix displaying the classification performance using PCA-LDA, RF, SVM, and CNN.

We set out to establish the Raman classification model to distinguish these two types of thyroid samples. The data in the range of 300–1800 cm^−1^ (fingerprint region) of the spectra were used for further analysis. We compared four ML diagnostic models, including PCA-LDA, RF, SVM, and CNN. As shown in Table 2, we used the PCA-LDA algorithm to cluster Raman spectra after dimensionality reduction, and its accuracy was only 62.08%. The PCA clustering results (Figure 6a) show much overlap between the benign and tumor groups, indicating unsatisfying diagnostic accuracy. Then, we tried RF analysis, with a higher accuracy of 77.33% and good specificity of 98.33%; but the sensitivity was only 56.33%. Similar results were observed for the SVM model, with 76.25% accuracy and 98.17% specificity, and a low sensitivity of 54.33%. For the above two models, the control group (benign samples) can mostly be accurately distinguished, but almost half of the malignant samples are misdiagnosed. We believe this is due to the intra-tumor heterogeneity of cancer tissues. Finally, we applied CNN. Although its ability to discriminate true negatives (i.e., specificity) was lower than RF and SVM, its sensitivity increased significantly to 87.8%. This led to a good classification of both groups with an accuracy of 88.1%. Figure 6 shows the confusion matrices for quantitative comparison on the test data of these models.

### 3.4. Performance Analysis and Interpretability of the Classification Model

**Figure 7:**
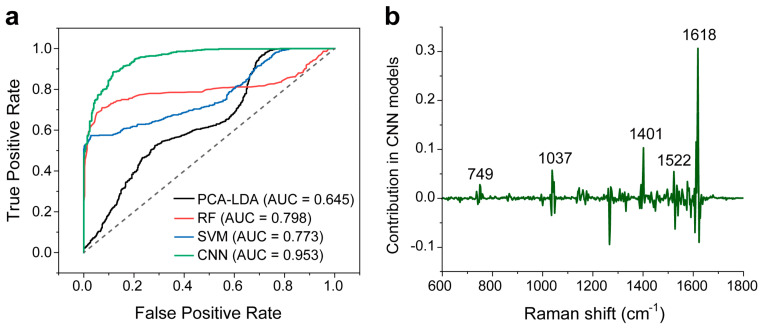
(**a**) ROC curves of dichotomous discriminations between the thyroid malignant and benign samples using the four models. The corresponding AUC for the CNN model is 0.953. The dotted line indicates performance under random events (0.5 AUC). (**b**) The characteristic Raman peak contribution in the CNN model.

We also compared the ROC curve of the four models (Figure 7a). The AUC values are 0.645, 0.798, and 0.773 for the PCA-LDA, RF, and SVM models, respectively. AUC values indicate the degree to which the model can distinguish between the two classes. The closer the AUC is to 1, the better the algorithm works. In comparison, the CNN model has a high AUC value of 0.935. This confirms its good classification capability.

Here, we would like to briefly explain the good classification performance of the CNN model. Figure 7b shows the main Raman peaks contributing to the CNN model. Positive peaks with values greater than 0 imply a positive contribution, and vice versa. We note that the characteristic main Raman peaks with positive contribution (749, 1037, and 1618 cm^−1^) are related to biochemical components that are listed in Table 1. Also, CNN provides additional peak information for biochemical assignments, e.g., 1401 cm^−1^ could be attributed to COO^−^ symmetric stretching, rich in fatty acids or liquids [7], and 1522 cm^−1^ is possibly due to C=C in plane vibrations of carotenoids [14]. These peaks indicated by CNN can be considered as the diagnostically significant Raman peaks.

## 4. Discussion

We would like to discuss three aspects:

1. The first is the advantage of using a quartz tube for the liquid SERS assay. We chose to measure through a quartz tube instead of a standard slide because our samples are in a liquid phase. The liquid SERS method improves the uniformity of sample measurement compared to spreading liquid samples on a microscope slide, where uneven spreading and drying can affect the SERS signal. In addition, the advantages of using the quartz tube include optical clarity and the low Raman signal background of quartz in the visible and near-infrared region, ensuring minimal interference with the Raman signal. A potential disadvantage is the alignment and focusing into the tube, which requires patience and experience. This was easily solved in our experiment: once the position of the quartz tube on the sample stage was optimized, we recorded and fixed this position for further tests.
2. The second advantage is the use of FNA washout fluids as SERS liquid biopsy samples. Unlike most previous SERS-based thyroid diagnostic reports that rely on serum or tissue samples, our study utilizes FNA washout fluids. While the understanding of their contents is still under investigation. During the puncture process, FNA samples contain cell clusters and interstitial fluid, which contains extracellular contents and substances released from damaged cells. The FNA samples are then transferred to the preservation solution, which stabilizes and preserves the original cell morphology. Typically, commercial preservation solutions contain formaldehyde, glutaraldehyde, and ethanol. These organic reagents could partially disrupt cell membranes, potentially releasing intracellular biomarkers such as metabolites and proteins into the solution. In our experiment, the FNA washout fluids were centrifuged and the bottom precipitates, consisting primarily of cells, were discarded. Therefore, it is reasonable to assume that the supernatants of the FNA washout fluids contain extracellular biomarkers and possibly some intracellular substances, which may be useful for SERS diagnosis. However, we acknowledge that further studies are needed to confirm this hypothesis.
3. Finally, we discuss the performance of CNN compared to PCA-LDA, SVM, and RF. In this work, the diagnostic accuracy is additionally contributed by the advanced deep learning model, CNN, whose robustness to variability within cancer tissues makes it particularly well suited for this type of diagnostic task. The superior performance of the CNN model can be attributed to the following reasons: CNN excels at handling high-dimensional data and automatically extracting hierarchical features from raw inputs, allowing it to capture intricate patterns and variations in Raman spectra. Unlike SVM and RF, which rely heavily on hand-crafted features and may struggle with tumor heterogeneity, CNNs can learn complex non-linear relationships directly from the data. This enables CNNs to effectively distinguish between subtle differences in spectral data, resulting in significantly higher sensitivity and overall accuracy.

## 5. Conclusions

In this study, we developed the label-free SERS liquid biopsy combined with the CNN model to achieve a rapid and accurate diagnosis of thyroid cancer. The biopsy samples were prepared using the mixture of Ag colloids and thyroid FNA washout fluids, dispersed in microfluidic quartz tubes for SERS measurements. We collected 36 clinical samples in total. Four classification models were established and compared: PCA-LDA, RF, SVM, and CNN. The results showed that the CNN algorithm is the most precise and stable, demonstrating a high accuracy of 88.1%, sensitivity of 87.8%, and a high AUC value of 0.953. Although its specificity is slightly less than the RF and SVM models, its capability to recognize true positives (sensitivity) is quite satisfying. Our ML-assisted SERS liquid biopsy is simple, convenient, and cost-effective. This study indicates that the label-free SERS technique combined with deep learning algorithms holds significant promise for the early identification and screening of thyroid cancer, offering valuable insights for the non-invasive and rapid clinical diagnosis.

## Figures and Tables

**Table 1:** Major vibrational band positions and their assignment to biochemical components.

SERS Peak (cm^−1^)	Assignments	References
747	Adenine, Cytochrome c	[13,14,25]
1038	τ(HCH)(CH_3_), τ(HCH)(CH_2_) collagen, phospholipids, phenylalanine	[14]
1163	ν(C-C), ν(C-N) of proteins and ν(C-C) of lipids, carotenoids	[13,14]
1396	CH-rocking, carbohydrates	[35]
1616	Amide I and lipids	[13]

**Table 2:** Performance comparisons (accuracy, sensitivity, specificity, and AUC).

	Accuracy	Sensitivity	Specificity	AUC
PCA-LDA	0.6208	0.4413	0.8283	0.645
RF	0.7733	0.5633	**0.9833**	0.798
SVM	0.7625	0.5433	0.9817	0.773
CNN	**0.8810**	**0.8780**	0.8840	**0.953**

Note: best results for each metric are highlighted in bold.

## Data Availability

Data supporting reported results can be found in the manuscript or from the authors at request.
